# Heterologous booster with inhaled adenovirus vector COVID-19 vaccine generated more neutralizing antibodies against different SARS-CoV-2 variants

**DOI:** 10.1080/22221751.2022.2132881

**Published:** 2022-11-04

**Authors:** Jiaying Zhong, Shuo Liu, Tingting Cui, Jingxin Li, Fengcai Zhu, Nanshan Zhong, Weijin Huang, Zhuxiang Zhao, Zhongfang Wang

**Affiliations:** a State Key Laboratory of Respiratory Disease & National Clinical Research Center for Respiratory Disease, Guangzhou Institute of Respiratory Health, the First Affiliated Hospital of Guangzhou Medical University, Guangzhou Medical University, Guangzhou, People’s Republic of China; b Department of Infectious Disease, Respiratory and Critical Care Medicine, Guangzhou First People’s Hospital, Guangzhou Medical University, Guangzhou, People’s Republic of China; c National Institutes for Food and Drug Control, NHC Key Laboratory of Research on Quality and Standardization of Biotech Products & NMPA Key Laboratory for Quality Research and Evaluation of Biological Products Beijing, Beijing, People’s Republic of China; d NHC Key Laboratory of Enteric Pathogenic Microbiology, Jiangsu Provincial Center for Disease Control and Prevention, Nanjing, People’s Republic of China; e Guangzhou Laboratory, Guangzhou, People’s Republic of China

**Keywords:** COVID-19, SARS-CoV-2, Omicron variant BA.5, vaccination strategies, adenovirus type 5 vector vaccine

## Abstract

The rapid widespread Omicron subvariant BA.5 of SARS-CoV-2 has become a potential imminent pandemic threat, but available vaccines lack high efficacy against this subvariant. Thus, it is urgent to find highly protective vaccination strategies within available SARS-CoV-2 vaccines. Here, by using a SARS-CoV-2 pseudovirus neutralization assay, we demonstrated that the aerosol inhalation of adenoviral vector COVID-19 vaccine after two dose of inactivated vaccine (I-I-Ad5) led to higher levels of neutralizing antibodies against D614G strain (2041.00[95% CI, 1243.00–3351.00] vs 249.00[149.10–415.70]), Omicron BA.2 (467.10[231.00–944.40] vs 72.21[39.31–132.70]), BA.2.12.1(348.5[180.3–673.4] vs 53.17[31.29–90.37]), BA.2.13 (410.40[190.70–883.3] vs 48.48[27.87–84.32]), and BA.5 (442.40 vs 56.08[35.14–89.51]) than three inactivated vaccine doses (I-I-I). Additionally, the level of neutralizing antibodies against BA.5 induced by I-I-Ad5 was 2.41-fold higher than those boosted by a third dose of RBD subunit vaccine (I-I-S) (*p* = 0.1308). The conventional virus neutralizing assay confirmed that I-I-Ad5 induced higher titre of neutralizing antibodies than I-I-I (116.80[84.51–161.5] vs 4.40[4.00–4.83]). In addition, I-I-Ad5 induced higher, but later, anti-RBD IgG and IgA in plasma than I-I-I. Our study verified that mucosal immunization with aerosol inhalation of adenoviral vector COVID-19 vaccine may be an effective strategy to control the probable wave of BA.5 pandemic in addition to two inactivated vaccines.

## Introduction

The rapid spread of the SARS-CoV-2 Omicron variants and its rapidly mutated subvariants have become a major global health concern. On 26 November 2021, Omicron BA.1 was firstly reported and quickly replaced the Delta strain as the primary strain of concern until 31 March 2022. On 29 March 2022, BA.2 emerged and became dominant within 8 weeks [1]. By the end of June 2022, new weekly cases were up by 32% in Southeast Asia, 33% in Europe, and 47% in the region comprising the Middle East, Central Asia, North Africa, and the Horn of Africa, as reported by the World Health Organization [2]. As of June 19, 2022, BA.5 accounted for 43% of Omicron cases worldwide, while BA.4 accounted for 12% [3]. More importantly, BA.5 was recognized to be the most transmissible coronavirus subvariant so far, and its mutations enable its escape from both natural infection- and vaccine-induced immunity, even among those who are exposed to the subvariant after recently recovering from an infection by the previous variant [4–6]. The United States Food and Drug Administration suggested the development of vaccine targets BA.4 and BA.5, and subvariant-specific vaccines are expected to be developed by the fall of 2022. However, the United States and Canada may be quickly overwhelmed by the rapid spread of BA.5 before the BA.5 variants specific vaccine were widely administrated. Owing to the high risk of BA.5-induced new wave of the COVID-19 pandemic in the United States, it is an urgent to identify or develop effective vaccination strategies using available vaccine technologies.

Inhalable vaccines based on adenovirus type 5 vector (Ad5) were designed to imitate the manner in which SARS-CoV-2 enters human bodies via the airways. Thus, it can induce a localized immune response targeting the mucosal surfaces to which pathogens attach instead of inducing a systematic immune response, which is facilitated by intramuscular vaccines [7–9]. Compared to intramuscular vaccines, which usually require low-temperature storage and trained health personnel for administration, inhalable vaccines can be administered through disposable devices with minimal storage requirements for mass vaccination. Therefore, inhalable vaccines can greatly reduce the cost for mass vaccination in developing countries [10]. Although inhaled adenovirus type 5 vector (Ad5 hereafter) COVID-19 vaccine has been reported to be safe and capable of inducing more neutralizing antibodies (NAbs) against the prototype based on a sequential vaccination strategy after two inactivated vaccines doses (I-I-Ad5) [11], it is not known whether this approach will be effective in response to rapidly emerging Omicron variants, especially under the imminent threat of Omicron BA.5.

In this study, we compared the immunological responses induced by three different vaccination strategies: low-dose or high-dose aerosolized inhaled Ad5 COVID-15 (I-I-Ad5) and intramuscular inactivated vaccine following two dose of intramuscular inactivated vaccines (I-I-I). We also investigated the level of NAbs against different Omicron subvariants BA.2, BA.2.12.1, BA.2.13 and BA.4/5 based on pseudoviral neutralizing experiments and found that I-I-Ad5 can effectively induce a higher level of NAbs against Omicron BA.5 than I-I-I, which elucidates better vaccine strategies for future vaccine programmes based on two previous inactivated vaccinations.

## Materials and methods

### Study cohort

Orally administered Ad5-nCoV vaccines contain a replication defective Ad5 vectored COVID-19 vaccine expressing a full-length spike gene from wild-type (WT) SARS-CoV-2, Wuhan-Hu-1 [12]. To assess whether heterologous immunization with aerosol inhalation induces robust antibody immune response, 150 participants who had received one booster dose at 6 months after two doses of inactivated vaccine were selected and divided into three groups to test three vaccination strategies. Group A comprised 50 participants with a median age of 41.62 years (41.62 ± 9.10). Group B comprised 50 participants with a median age of 40.90 years (40.90 ± 9.77). Group C comprised 50 participants with a median age of 42.12 years (42.12 ± 8.56) (Table 1). Group A (low I-I-Ad5) was boosted with low-dose aerosolized Ad5-nCoV (2 × 10^10^ virus particle/ml in 100 µl). Group B (high I-I-Ad5) was boosted with the high-dose aerosolized Ad5-nCoV (2 × 10^10^ virus particle/ml in 200 µl). Group C was boosted with a homologous third dose of inactivated vaccine (CoronaVac, Sinovac Biotech) by intramuscular injection. Plasma samples were obtained at 0, 7, 14, and 28 days after the third dose was administered. Additionally, we collected samples from vaccinees who were boosted with the RBD protein-subunit vaccine (ZF2001, Zhifei Biological) (I-I-S) 14 days after immunization. Written informed consent was obtained from each participant before inclusion in the study. This study was approved and supervised by the GMUH Ethics Committee (No. 2021-78).

### Enzyme-linked immunosorbent assay (ELISA)

Whole blood samples were centrifugated at 800 g for 10 min to obtain the plasma samples. A direct ELISA was conducted against the SARS-CoV-2 S protein RBD domain using a commercial ELISA kit (Darui Biotechnology, Guangzhou, China) to detect specific plasma anti-RBD IgG. A sample with a high anti-RBD titre was used as the standard sample with an arbitrary unit (unit/mL) of 500. Next, a two-fold serial dilution of the standard sample was performed at each instance to evaluate other plasma samples according to the manufacturer’s instructions. We used a washing buffer as the blank control. Absorbance was measured at 450 nM by a Multiskan GO microplate spectrophotometer (Thermo Fisher Scientific, Waltham, MA, USA). Data were analyzed using a standard curve with a log-logistic model.

**Table 1.** Characteristics of participants.

Characteristics	low I-I-Ad5 (*n* = 50)	high I-I-Ad5 (*n* = 50)	I-I-I (*n* = 50)	I-I-S (*n* = 14)
Gender (*n*, %)				
Male	25 (50%)	19 (38%)	23 (46%)	2 (14.29%)
Female	25 (50%)	31 (62%)	27 (54%)	12 (85.71%)
age (means ± SD)	41.62 ± 9.10	40.90 ± 9.77	42.12 ± 8.56	25.86 ± 2.82^#,*^

^#^ and * indicate significant differences(*p* < 0.0001) compared with the other three groups.

### SARS-CoV-2 conventional virus neutralization test

Plasma neutralization activity was evaluated using a cytopathic (CPE)-based assay as previously described [13]. Plasma samples were tested at an initial dilution of 1:8 and then diluted in eight two-fold steps. All samples were mixed with a SARS-CoV-2 Wuhan-1 Omicron viral solution that contained 100 TCID50 of the virus and incubated in 37°C and 5% CO_2_ for 2 h. Next, the virus–plasma mixture was added to a 96-well plate containing 1.2 × 10^4^ Vero E6 cells. The plates were incubated for 4 days at 37°C in a humidified environment with 5% CO2 and examined for CPE by the Celigo Imaging Cytometer (Nexcelom Bioscience, Lawrence, MA, USA). The absence or presence of CPE was defined by comparing each well a positive control (plasma sample showing high SARS-CoV-2 neutralizing activity in infected Vero E6 cells) and a negative control (human serum sample negative for SARS-CoV-2 in ELISA and neutralization assay and Vero E6 cells alone). We defined neutralizing antibody titres less than the detection limit dilution as NAbs titre of 50% inhibitory dilution (EC50) = 4.

### Cell-based competitive ELISA (ccELISA) for NAb cluster detection

Mouse NAbs clones 13G2 and 08B3 were purified using a His-Trap Protein G column (GE Healthcare Life Sciences, USA), and the purified NAbs were conjugated to horseradish peroxidase (HRP) using the EZ-Link Plus Activated Peroxidase kit (Thermo Scientific, USA) according to the manufacturer’s instructions. Cluster-targeting NAbs were measured using an in-house ccELISA. Previous studies from our laboratory have described the method for producing A549 cells stably expressing SARS-CoV-2 [14]. Cells were seeded in 96-well plates at 2 × 10^4^ cells/well. At 24 h after seeding, cells were fixed with 4% paraformaldehyde at room temperature for 20 min and further blocked for 2 h with phosphate-buffered saline solution Tween containing 3% bovine serum albumin at 37°C. Next, 50 μL of each diluted serum sample was mixed with 50 μL of diluted HRP-13G2 or HRP-08B3, added to the A549 cells, and incubated at 37°C for 1 h. After extensive washing, 100 μL of tetramethylbenzidine stabilized chromogen (Invitrogen, USA) was added. Cells were then incubated at 37°C for 10 min, followed by treatment with 50 μL of stopping solution (R&D Systems, USA). Absorbance was measured at 450 nm using a Multiskan GO microplate spectrophotometer (Thermo Fisher Scientific, Waltham, MA, USA). Furthermore, to standardize the assay across the plate and quantify the results, an arbitrary titre was applied in each tested sample based on the standard curve drawn from a dilution series of sera with known competition rates against 13G2 and 08B3 clones.

### Pseudovirus-based neutralization assay

SARS-CoV-2 and Omicron variants were examined and deemed representative of the original SARS-CoV-2 strain and emerging variants with mutations in the spike protein. Neutralization was measured by reducing the luciferase gene expression as previously described for the HIV pseudovirus neutralization assay [15]. The EC50 was defined as the serum dilution at which the relative light units (RLUs) were reduced by 50% compared to the virus control wells (virus + cells) after subtracting background RLUs in the control groups with cells only. Briefly, the pseudovirus was incubated with serial dilutions of the test samples (six dilutions in a three-fold stepwise manner) in duplicate for 1 h at 37°C together with the virus and cell control wells in hexaplicate. Freshly trypsinized cells were added to each well. Following 24 h of incubation at 5% CO_2_ and 37°C, luminescence was measured, as described in the pseudovirus titration section. EC50 values were calculated by non-linear regression, that is, log (inhibitor) vs. response (four parameters), using GraphPad Prism (version 6, San Diego, CA, USA).

### Indirect chemiluminescence detected SARS-CoV-2 IgA

We used the indirect chemiluminescence method to detect SARS-CoV-2-IgA specific antibodies in human serum samples. The automated analytical instrument Axceed 260 and accompanying immunoassay test kits were used for the clinical chemiluminescence immunoassay (Tianjin Bioscience Diagnostic Technology Co., Ltd.). Axceed 260 automatically conducted the following tasks: adding reagents, adding samples, adding magnetic beads, incubation reaction and mixing, cleaning magnetic beads, adding substrate and mixing, and reading RLUs. Obtained RLUs were then converted to final titre readouts in titre units of S/CO. The IgA test kit used in this study is called the Diagnostic Kit for Novel Coronavirus (2019-nCoV) IgA Antibody (Magnetic particle CLIA) [16].

### Statistical analyses

Statistical analyses were performed by using GraphPad Prism software. The Mann–Whitney test was conducted to compare the central tendencies of the two groups (mean or median). Antibody responses were reported as geometric mean titres (GMTs) with a 95% confidence interval (CI). Wilcoxon rank-sum test was used to compare paired continuous variables that were not normally distributed.

## Result

### Third dose of aerosol inhalation of Ad5 COVID-19 vaccine induces superior anti-spike antibody response to intramuscular inactivated vaccination

**Figure 1.**
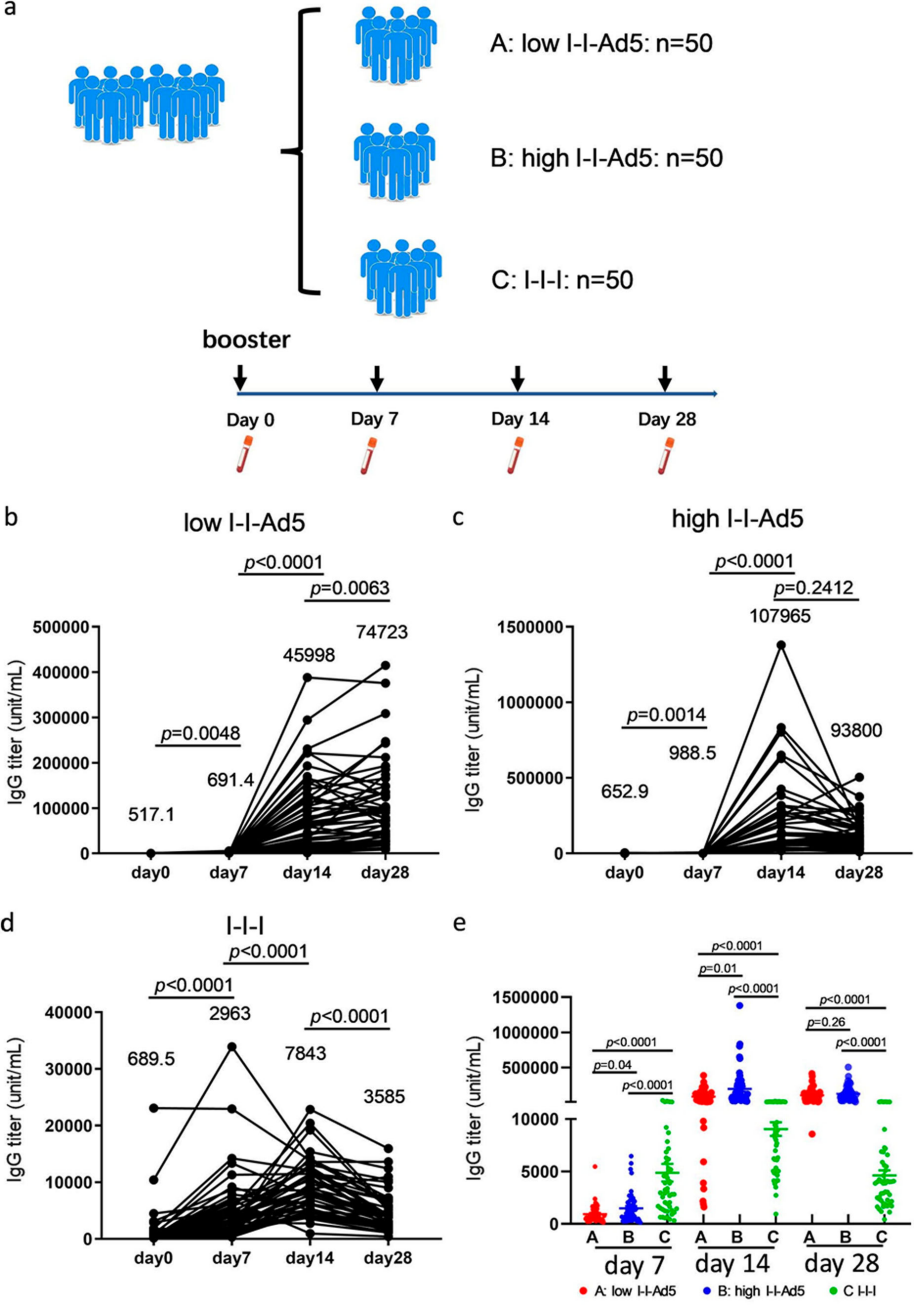
(a) Grouping information and timing of plasma sample acquisition. Groups A, B, and C comprised vaccinees who received a heterologous booster vaccination with a low-dose aerosolized Ad5-nCoV (low I-I-Ad5, *n* = 50), heterologous booster vaccination with a high dose aerosolized Ad5-nCoV (high I-I-Ad5, *n* = 50), and a homologous booster of inactivated vaccine (C group: I-I-I, *n* = 50), respectively. Plasma samples were obtained at 0, 7, 14, and 28 days after third dose administration. (b–d) Kinetics of SARS-CoV-2-RBD IgG in three groups after third dose administration. (e) Comparison of SARS-CoV-2-RBD IgG titre among all groups at multiple time points. The IgG titre in each group is shown as the GMT at the top of each panel. *p*-values for group differences are shown above each group.

To assess whether the booster of inhalable Ad5 vaccines can effectively induce robust antibody immune responses, 150 vaccinees who had received one booster dose 6 months after the initial two doses of inactivated vaccine were recruited in this study and divided into three groups. Groups A, B, and C were boosted with low-dose aerosolized Ad5-COVID-19 vaccine (low I-I-Ad5), high-dose aerosolized Ad5 COVID-19 vaccine (high I-I-Ad5), and a homologous third dose of inactivated vaccine (CoronaVac) by intramuscular injection (I-I-I), respectively. The plasma samples were collected at days 0, 7, 14 and 28 after the third dose administration (Figure 1(a)). We then detected the kinetics of antibodies against the SARS-CoV-2 RBD by ELISA. Group A showed a significant increase in RBD-IgG levels at day 14 after vaccination (*p* < 0.001), which consistently increased until day 28 (Figure 1(b)). Groups B and C showed a peak in antibody response at day 14 after immunization, which declined by day 28 (Figure 1(c,d)).

The anti-RBD IgG levels of group C were much higher (2963 [95% CI 2219–3956]) than those of groups A (691.4[559.3–854.8], *p* = 0.0001) and B (988.5[755.2–1294], *p* = 0.0001) at day 7 after immunization (Figure 1(e)), indicating that considerable anti-RBD IgG antibodies were rapidly induced after the homologous inactivated vaccine booster. The anti-RBD IgG levels of – only group C peaked at day 14 and declined thereafter. In contrast, the anti-RBD IgG of Groups A (45,998[30,859–68,562], *p* = 0.0001) and B (107,965[79,992–145,721], *p* = 0.0001) far exceeded those of Group C at day 14 (7843[6656–9242]) (Figure 1(e)). Although the antibody titre decreased at day 28 for all groups, those of Groups A and B were still higher than that of Group C (*p* < 0.0001). Our results suggest that a heterologous booster of inhaled Ad5 vaccine induced higher level of RBD-IgG antibodies than that of a homologous inactivated booster, but the improved heterologous response occurs later than the homologous response.

### Aerosol inhalation of Ad5 COVID-19 vaccine induced more NAbs against prototype and Omicron variants

**Figure 2.**
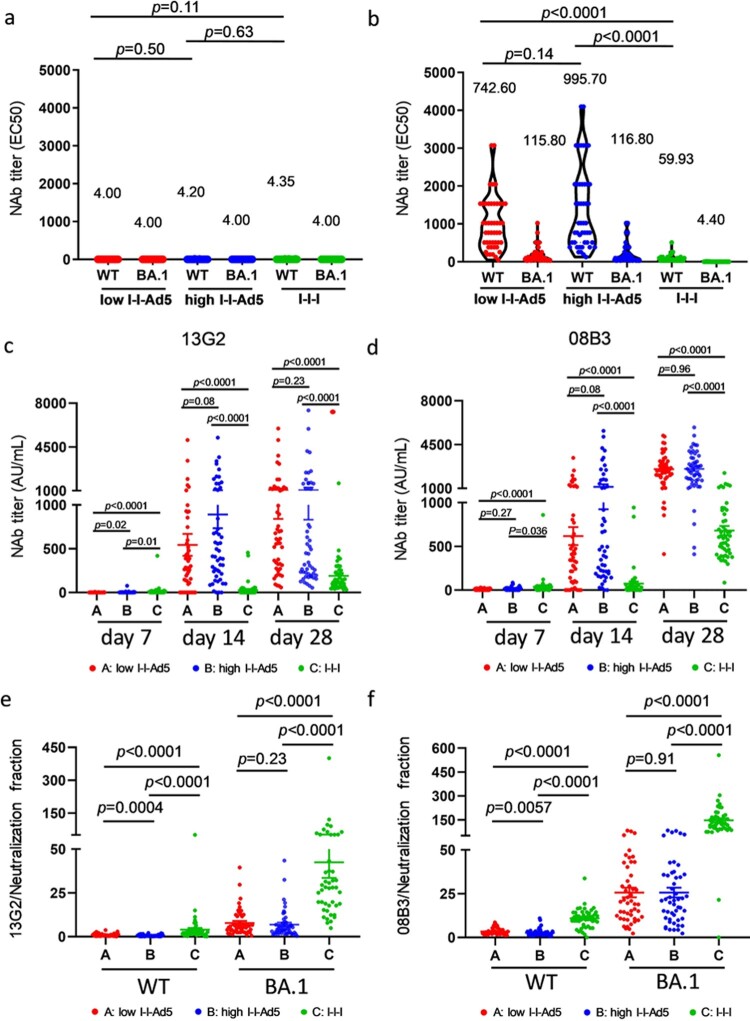
Comparison of neutralizing antibody titre among all groups. (a) Levels of NAbs against WT and Omicron BA.1 before immunization. (b) Neutralizing antibody titres against SARS-CoV-2 WT and Omicron variants BA.1 strains in all groups at day 28. The NAbs titre in each group is shown as the GMT at the top of each panel, and factor differences from WT to Omicron BA.1 subvariants are indicated. *p*-values for group differences are shown above each group. (c) Comparison of 13G2 neutralizing titre among all groups at 7, 14 and 28 days after third dose administration. (d) Comparison of 08B3 neutralizing titre among all groups at 7, 14, and 28 days after third dose administration. Portion of NAbs represented by monoclonal antibodies 13G2 (e) and 08B3 (f) compared to neutralizing antibody for all groups at day 28. *p*-values for group differences are shown above each group.

Using a SARS-CoV-2 conventional virus neutralization test, we detected NAbs against SARS-CoV-2 WT and Omicron BA.1 among three groups on days 0 and 28. Neutralizing antibody levels against SARS-CoV-2 were low before booster immunization (Figure 2(a)). Results showed that Groups A (742.6[576.3–956.8], *p* = 0.0001) and B (995.7[770.2–1287], *p* = 0.001) exhibited significantly higher NAbs against WT than Group C (59.93[48.63–73.86]) (Figure 2(b)), and NAbs against Omicron variants BA.1 in Groups A (115.8[48.63–73.86], *p* = 0.001) and B (116.8[88.57–151.3], *p* = 0.001) were also significantly higher than those in Group C (4.40[4.00–4.84]) (Figure 2(b)). Furthermore, Groups A and B exhibited similar NAbs against WT and Omicron BA.1.

To study the composition of NAbs induced by all groups, competitive ELISA was performed to measure the levels of two NAbs clusters targeted or shared with the epitopes of two NAbs clones, 13G2 and 08B3, as conducted in our previous study [17]. Our results showed that the NAbs of Groups A and B represented by 13G2 and 08B3 clones are much higher than those of Group C (*p* < 0.0001) (Figure 2(c,d)). The ratio between levels of individual NAbs (represented by 13G2 and 08B3) and total NAbs, we found that the responses of Groups A and B differed significantly from that of Group C (Figure 2(d,e)).

### I-I-Ad5 effectively induced more NAbs against emerging BA.5 variant

**Figure 3.**
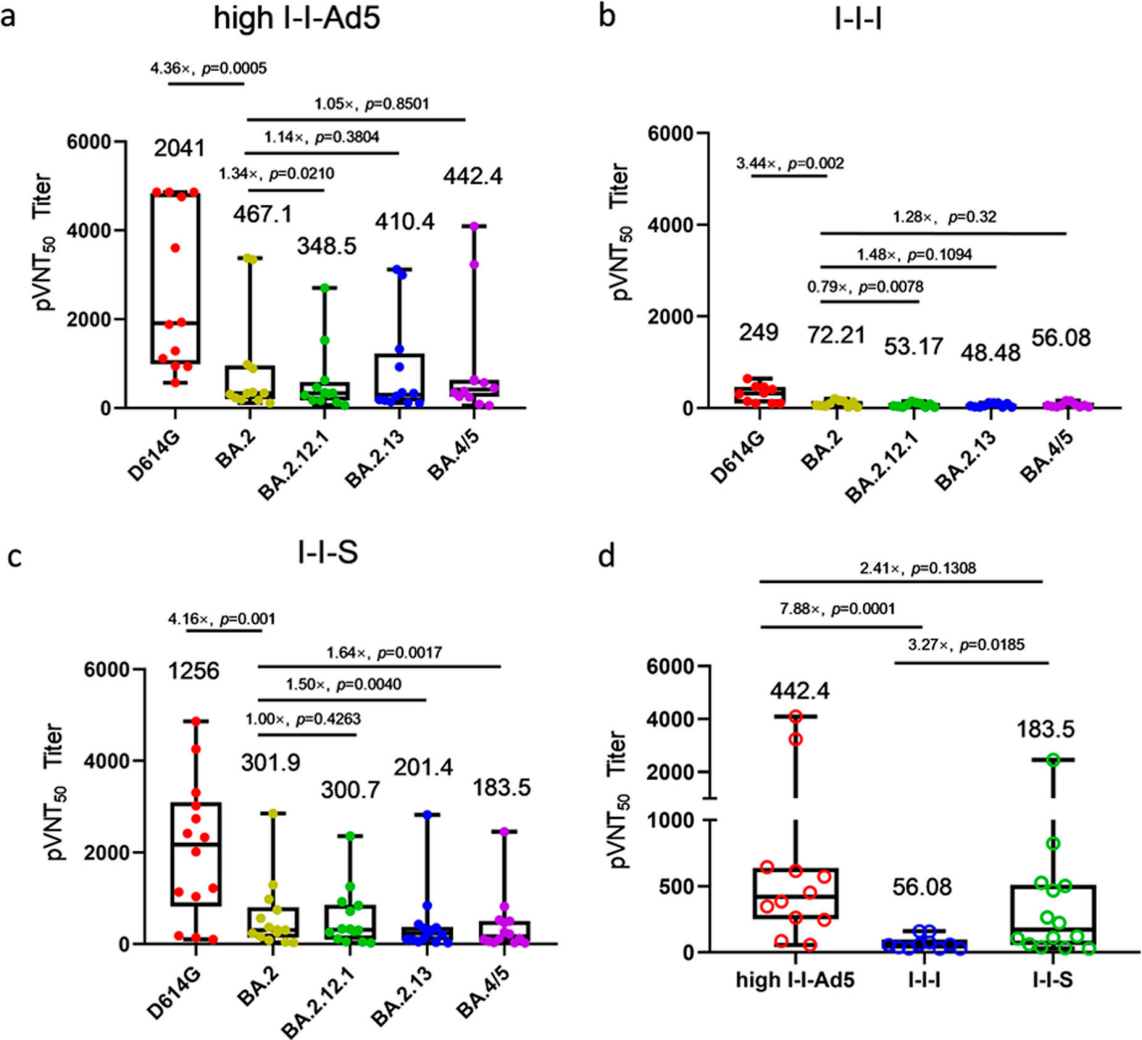
The 50% pseudovirus neutralization titre (pVNT50) against the listed D614G isolate and Omicron subvariants BA.2, BA.2.12.1, BA.2.3, BA.4, and BA.5. Samples were obtained approximately 14 days after third dose administration. (a) Neutralizing titres of high-dose I-I-Ad5 against the D614G isolate and Omicron subvariants (*n* = 12). (b) Neutralizing titres of I-I-I against the D614G isolate and Omicron subvariants (*n* = 10). (c) Neutralizing titres of vaccinees boosted with the RBD protein-subunit vaccine (ZF2001) (I-I-S) against the D614G isolate and Omicron subvariants (*n* = 14). (d) Neutralization antibody titre levels against BA.4/5 were compared across the three immunization strategies. The pVNT50 in each group is shown as the GMT at the top of each panel, and factor differences from other subvariants are indicated. Box plot explanation: Upper horizontal line of box, 75th percentile; lower horizontal line of box, 25th percentile; horizontal bar within box, median; upper horizontal bar outside box, maximum; lower horizontal bar outside box, minimum.

To evaluate whether I-I-Ad5 can effectively induce protective immunity against the emerging BA.5 strain, a SARS-CoV-2 pseudovirus neutralization assay was carried out to assess plasma neutralizing antibody titres against the SARS-CoV-2 prototype (D614G) isolate and Omicron subvariants BA.2, BA.2.12.1, BA.2.13, and BA.4/5. The plasma samples were obtained at day 14 after administration of the third dose, and three groups were considered for evaluation (high-dose I-I-Ad5 (group B), I-I-I (group C), and group boosted with the RBD protein-subunit vaccine (ZF2001) (I-I-S)). In all three groups, the titre against Omicron subvariants were significantly lower than the D614G strain by 3–4-fold (*p* < 0.01) (Figure 3(a–c)). This result indicates that Omicron escapes D614G-targeting vaccine-induced neutralizing antibody responses, and the protection afforded by vaccines currently designed for SARS-CoV-2 prototype S protein or RBD domain is less effective against these variants. Moreover, the neutralizing antibody responses against BA.4/5 were 1.28-fold (*p* = 0.32) and 1.64-fold (*p* = 0.0017) lower than those against BA.2 in I-I-I and I-I-S groups, while the neutralizing antibody response against BA.4/5 in the I-I-Ad5 group was comparable to that against Omicron BA.2 (467.10 [231.0–944.40] vs 442.4[201.8–971.50]). Among the three groups, I-I-Ad5 exhibited 2.41-fold and 7.88-fold higher NAbs against BA.4/5 (442.80[201.8–971.5]) than I-I-S (183.5[85.6–393.6], *p* = 0.1308) and I-I-I (56.08[35.14–89.51], *p* = 0.0001) (Figure 3(d)), respectively.

### Inhaled aerosolized immunization induced higher level of RBD-specific IgA in plasma than those boosted with inactivated vaccine

**Figure 4.**
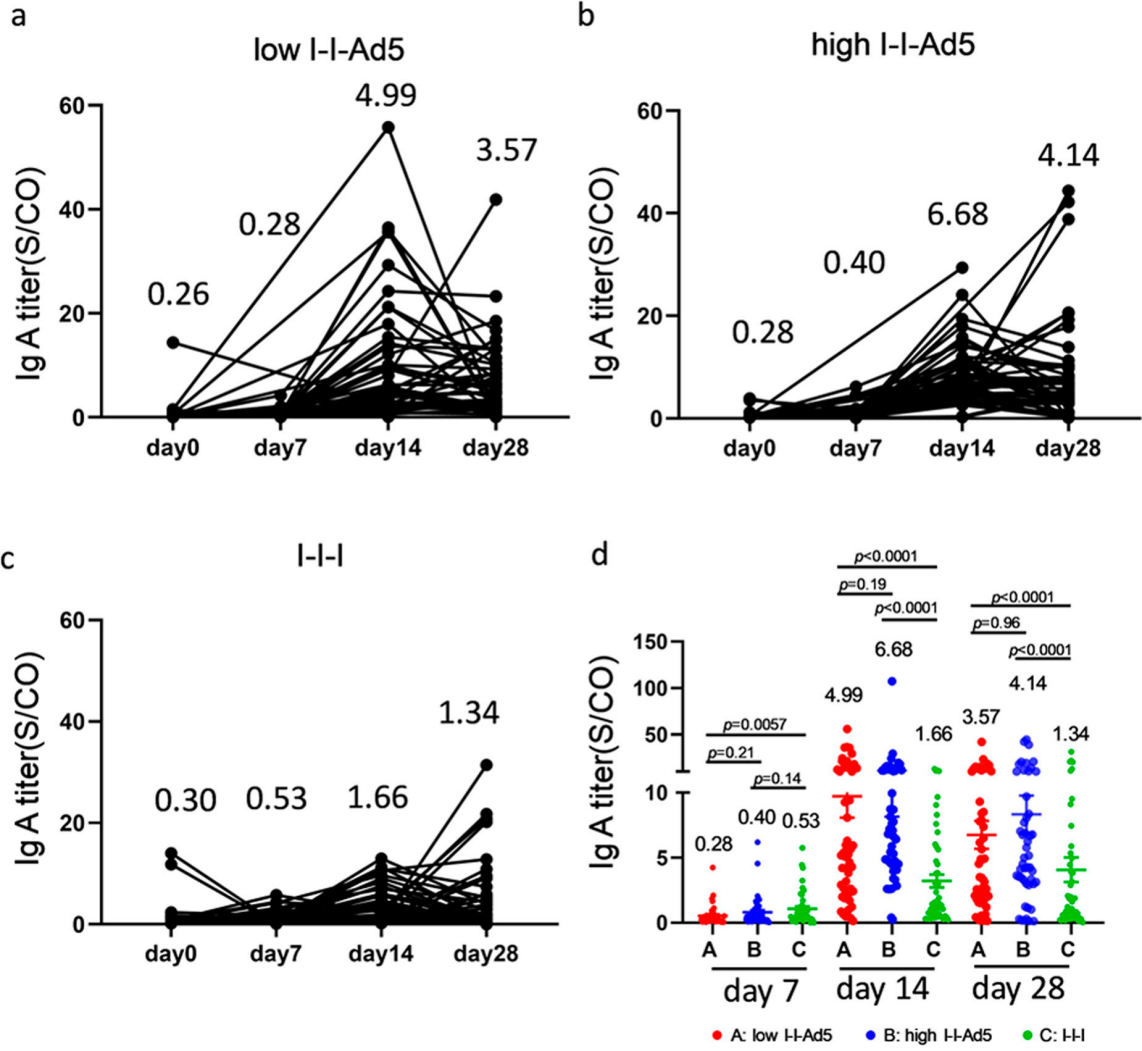
Inhaled aerosolized immunization induced higher levels of RBD-specific IgA in plasma than inactivated vaccine booster. (a–c) Total kinetics of SARS-CoV-2-IgA antibody titre at 0, 7, 14, and 28 days after third doe administration (*n* = 50). (d) Comparison of IgA titre between groups at each time point. IgA titre in each group is shown as the GMT at the top of each panel. *p*-values for group differences are shown above each group.

Aerosolized immunization of the Ad5 COVID-19 vaccine has the advantage of inducing local mucosal IgA antibodies at the infection entry site, which may then affect the antibody count in blood. Therefore, we measured RBD-specific IgA levels at days 0, 7, 14, and 28 days after the third dose administration. Our results showed that IgA peaked at day 14 and decreased at day 28 (Figure 4(a–c)). The IgA responses exhibited in Groups A (4.99[3.465–7.173], *p* = 0.001) and B (6.68[5.10–8.75], *p* = 0.001) were significantly higher than that for Group C (1.66[1.17-2.35]) by day 14 after third dose administration. Interestingly, IgA levels in the high-dose nasal inhalation group were higher than the low-dose group (1.33-fold, *p* = 0.19), suggesting that IgA production was related to the inhaled antigen concentration.

## Discussion

With R0 = 18.6 and excellent immune escape capacity from vaccine- and natural infection–induced immunity, BA.5 presents a significant obstacle in the timely development of efficacious vaccines. The FDA estimates that Omicron-specific boosters from Pfizer and Moderna will become available in early to mid-fall of 2022, but BA.5 infections are already approaching more than 50% in US and 70% in Canada [18]. It is crucial to employ different immunization strategies to enhance the level of the NAbs as Omicron subvariant BA.5 is considered to have a high risk for inducing the next wave of the COVID-19 pandemic. Our study clearly showed that inhaled aerosolized Ad5 COVID-19 vaccine followed after two dose of inactivated vaccine may induce higher neutralizing antibody levels against BA.5, suggesting that its use in existing vaccine regimens in China exhibits significant potential for preventing emerging Omicron-induced pandemic waves.

High and low doses of inhaled aerosolized Ad5 COVID-19 vaccine did not induce significantly different levels of anti-RBD IgG antibodies or NAbs production, indicating that the limited replication of high and low dose of Adenovirus can lead to similar antigen exposure and thus similar antibody production. However, whether SARS-CoV-2 spike protein expressed by different adenoviral vector doses can lead to saturated expression, requires further investigation. Interestingly, high- and low-dose Ad5 COVID-19 vaccines induced fewer NAbs represented by clone 08B3 and less anti-RBD IgG than the inactivated vaccine did, showing less S antigen production by the Ad5 vaccine than the amount of S proteins carried by non-replicative inactivated virus particle during the first 7 days.

Additionally, the mucosal delivery route can elicit local mucosal immune responses, including IgA and virus-specific T cell response [7,11,19]. Recent animal studies demonstrated that mucosal immunization can control viral replication in the respiratory tract [20,21]. The primary limitation of this study is that we measured the level of IgA in blood rather than at the infection site because at the beginning of this clinical experiments and illustrated in the ethics application, IgA test in nasal wash or induced sputum are not included. Nevertheless, inhaled aerosolized Ad5 COVID-19 vaccine led to increased IgA production compared to the intramuscular inactivated vaccine in a dose-dependent manner, indicating that higher levels of mucosal IgA may be induced by the Ad5 vaccine and subsequently increase IgA levels in the blood. Overall, our findings strengthen recent evidence that targeted mucosal immunization may be an effective strategy for controlling the expecting spike in BA.5-induced infections in the COVID-19 pandemic based on two inactivated vaccines.
